# Imaging Findings of Pseudo-occlusion of Common Iliac Artery: Pitfall of Time-resolved Contrast-enhanced Magnetic Resonance Angiography

**DOI:** 10.7759/cureus.7252

**Published:** 2020-03-12

**Authors:** Hiroyuki Tokue, Hideo Morita

**Affiliations:** 1 Department of Diagnostic and Interventional Radiology, Gunma University Hospital, Gunma, JPN

**Keywords:** uterine fibroids, uterine artery embolization, time-resolved contrast-enhanced magnetic resonance angiography

## Abstract

Time-resolved contrast-enhanced magnetic resonance angiography (TRC-MRA) is a contrast-enhanced MRA technique commonly used for the qualitative hemodynamic assessment in digital subtraction angiography. TRC-MRA is a reliable method that seldom shows flow-related false findings which are sometimes observed on noncontrast or single-phase contrast-enhanced MRA techniques. Here we present a case of a patient with large fibroids that showed pseudo-occlusion of common iliac artery in TRC-MRA. Flow alternation can take place depending on patient posture and positioning status, especially in cases with large pelvic mass lesions. This is the first report of false image findings related to pelvic mass observed on TRC-MRA. For patient preparation, coil setting position is important and physicians should be familiar with the potential risk of transient arterial luminal stenosis or even occlusion in patients with large pelvic mass.

## Introduction

Time-resolved contrast-enhanced magnetic resonance angiography (TRC-MRA) is a contrast-enhanced MRA technique commonly used for the qualitative hemodynamic assessment in digital subtraction angiography. There are many contrast-enhanced MRA techniques, and TRC-MRA is one of them. In this technique, changes in enhancement are caught continually as changes of blood flow by repetition data sampling of the same position under rapid contrast agent injection. The signal decay due to velocity, direction, or pattern of blood flow is limited because of dominant T1 shortening effect. Continuous, repeated data sampling can readily distinguish the false images due to temporal flow abnormality and true images.

TRC-MRA is a reliable method that seldom shows flow-related false findings which are sometimes observed on noncontrast or single-phase contrast-enhanced MRA techniques [1]. Here we present a case of a patient with large fibroids that showed pseudo-occlusion of common iliac artery in TRC-MRA. We considered the reasons of causative mechanisms.

## Case presentation

**Figure 1:**
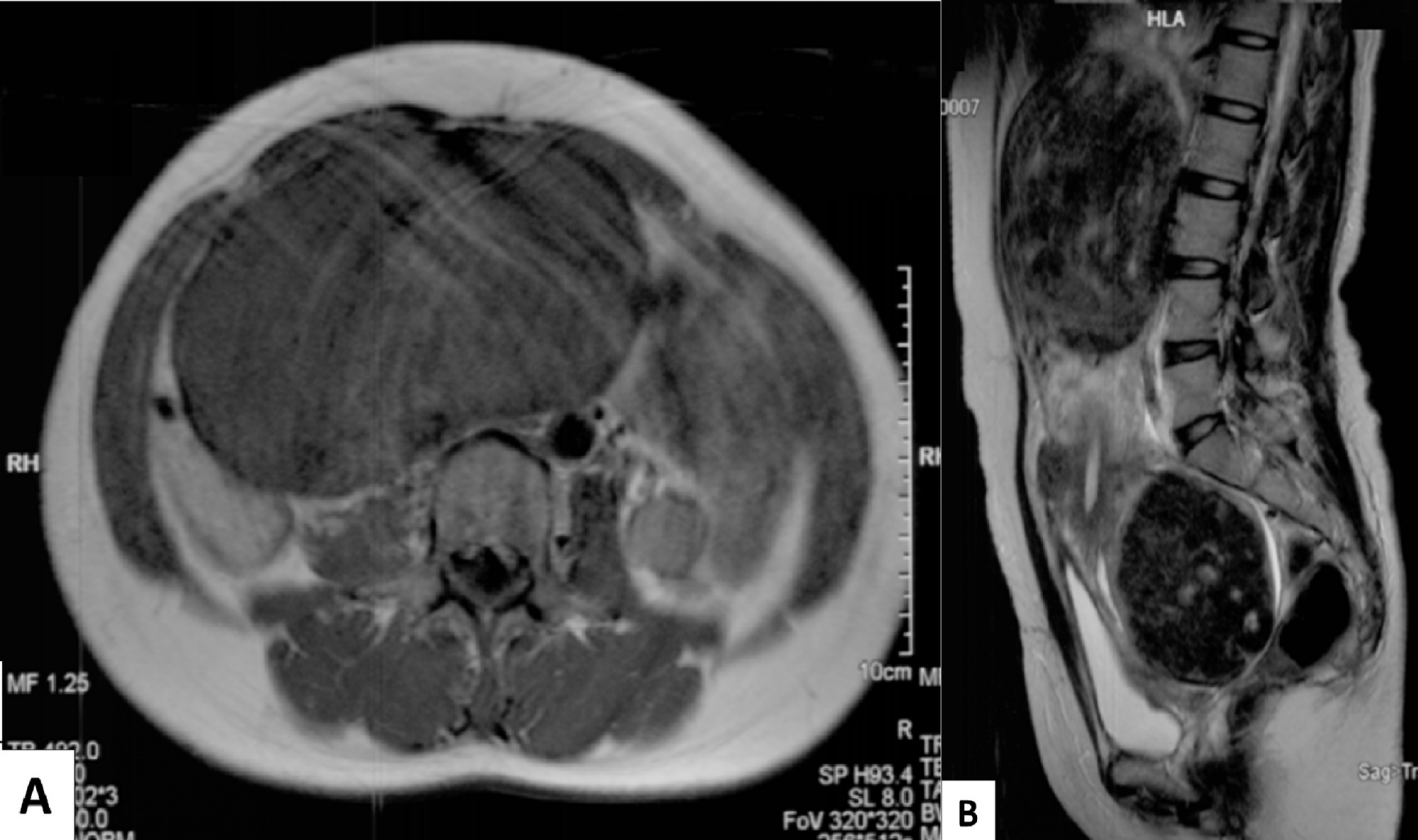
MRI before uterine artery embolization. (A) Axial image shows massive leiomyoma of intermediate signal intensity on T1-weighted image. (B) Sagittal T2-weighted image. Enlarged uterus compressed and displaced the bladder and bowel loops.

A 41-year-old Japanese woman was referred to our hospital with excessive menstruation and pressure symptoms due to large fibroid tumors. Her lower abdomen showed slight bulging, and a palpable mass was noted. She had no past history of coagulation disorder. Contrast-enhanced MRI showed large intramural fibroids at the uterine cervix and fundus of intermediate signal intensity on T1-weighted images (T1-WIs) and heterogeneously low to high signal intensity on T2-WIs. T1-WIs was acquired using a breath-hold gradient-echo sequence with the following parameters: repetition time (TR)/echo time (TE), 750/10, ms; matrix, 320 ×  192 pixels; field of view (FOV), 400 mm; slice thickness, 5.0 mm. T2WI was acquired under breath-holding and the parameters were as follows: TR/TE, 2,250/90 ms; matrix, 320 × 192 pixels; FOV, 400 mm; slice thickness, 5.0 mm. There were also many small intramural fibroids with a similar signal intensity pattern. The size of uterine fibroids in the pelvis was 13x8x11 cm. The enlarged uterus due to fibroids severely compressed the bladder and bowel loops (Figure 1). We supposed that uterine fibroids were the cause of her symptoms. Thus, after written informed consent, uterine artery embolization (UAE) was performed to relieve her symptoms. Under local anesthesia, right femoral arterial puncture and insertion of 5-French vascular sheaths were performed.

In arteriography, meandering and developed uterine arteries were shown. Embolization was performed with gelatin sponge particles into the bilateral uterine arteries with 2.0-French microcatheter until there was stasis of contrast [2]. After UAE, the main branches of bilateral uterine arteries were disappeared and she was discharged five days after the procedure without complications.

One month later, MRI was performed. We evaluated the vascularity of the bilateral uterine arteries with the time-resolved imaging of contrast kinetics (TRICKS) technique [3]. The TRICKS sequence parameters were TR/TE, 5.4/1.8 ms; flip angle, 45°; FOV, 28 cm; matrix, 320 × 192 pixels, and slice thickness, 1.2 mm. The temporal resolution was three to four seconds for each phase, and a total of 15 phases were acquired within two minutes. Following the acquisition of a mask, 20 mL of gadolinium-based contrast agent was administered intravenously at 3 mL per second by a pump injector followed by a 50-mL saline bolus. All scans were performed on a Signa 1.5T scanner (GE Healthcare, Milwaukee, Wisconsin) by using a cardiac phased array coil.

**Figure 2:**
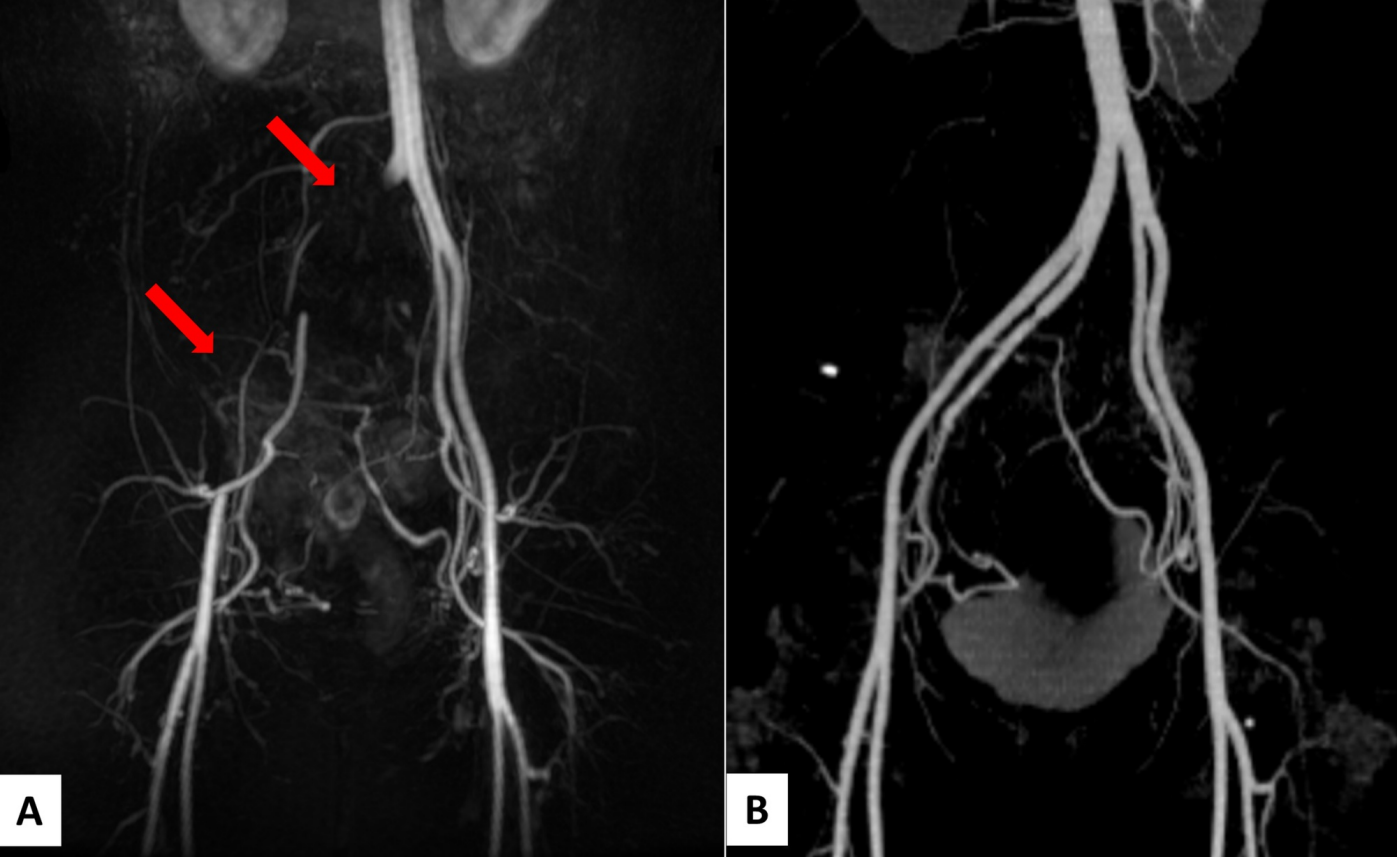
Time-resolved contrast-enhanced magnetic resonance angiography (MRA) images and CT angiography one month later. (A) Maximum intensity projection (MIP) of time-resolved contrast-enhanced MRA images show lack of enhancement of right common iliac artery and proximal external iliac artery (arrows). Distal arterial branches are visualized via internal iliac circulation. (B) MIP of CT angiography shows normal opacification of bilateral iliac and femoral arteries, and no vascular wall abnormality is recognized.

On TRC-MRA, the right common iliac artery and proximal external iliac artery were not observed and distal arterial branches were visualized via internal iliac circulation (Figure 2A). These imaging findings were suggestive of occlusion of right common and external iliac artery. The proximal external iliac arterial lumen showed a moderately high signal on T1-WIs and T2-WIs. And, flow void signal was not observed. Postcontrast fat-suppressed T1-WIs showed lack of enhancement of all uterine fibroids after UAE [4]. After the examinations, weak pulses of both the right inguinal artery and dorsal pedal arteries could be felt, without pain, change in skin color, or intermittent claudication. She had never felt edema or discomfort in her right foot, after the UAE. Enhanced CT was performed immediately after the MR examination to confirm the presence of arterial occlusion.However, opacification of the bilateral arteries from pelvis to femur was shown on the CT angiography, and vascular wall abnormality was not recognized (Figure 2B). The arterial occlusion suspected on MRI was confirmed to be a false imaging. No treatment was required, and there were no symptoms after the examinations.

## Discussion

TRC-MRA is one of a widely used technique of pelvic MRA. Recently, the progress of fast MRI techniques makes it possible to collect three-dimensional (3D) data in a matter of seconds, and use of TRC-MRA, which repeatedly collects 3D data sets and provides dynamic 3D vascular images, has become practical on a clinical level.

TRC-MRA is considered one of the most reliable methods for blood imaging [1,5]. Compared to non-contrast techniques such as 2D time-of-flight or 3D electrocardiographic-gated fast spin echo MRA, this technique largely depends on contrast enhancement of blood in the vascular lumen, and false findings induced by flow pattern are extremely rare. The timing of contrast injection and image acquisition do not affect image quality, as each vessel is imaged several times repeatedly at different time frames.

**Figure 3:**
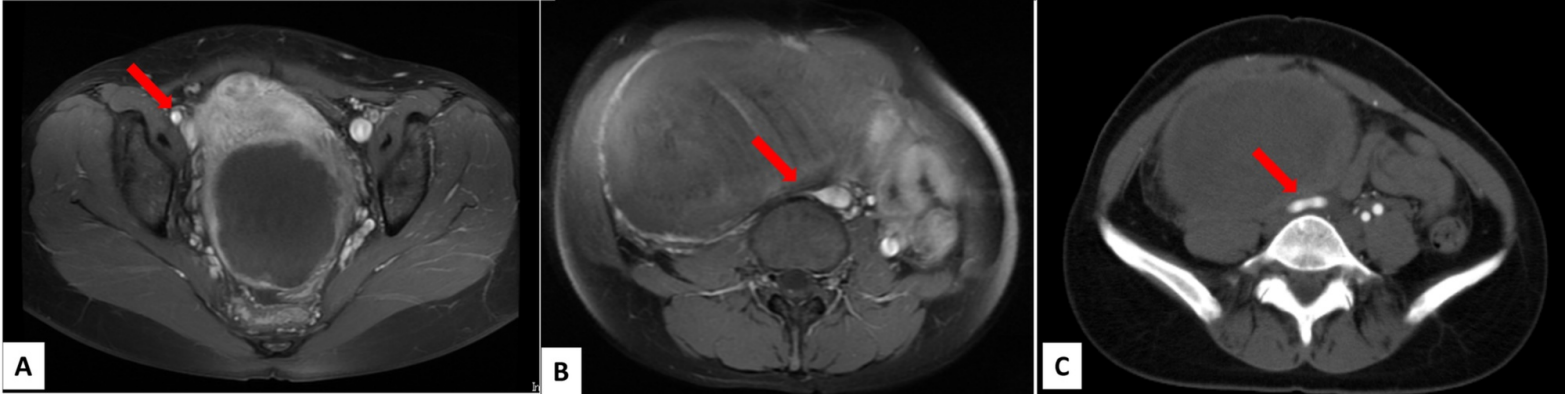
Contrast-enhanced T1-weighted images and contrast-enhanced CT images one month later. (A) Contrast-enhanced T1-weighted images show enhancement of right external iliac artery (arrow). (B) Right common iliac artery is flattened and unclear (arrow). (C) Contrast-enhanced CT images show patency of right common iliac artery.

This is the first report of false image findings related to pelvic mass observed on TRC-MRA. In our case, TRC-MRA showed lack of enhancement from the right common iliac artery to external iliac artery in multiple time frames, and right lower extremity arteries were supplied through collateral vessels from the left internal iliac artery. High signal intensity of the common iliac artery lumen on T1-WIs and T2-WIs seemed consistent with recently developed thrombus. However, inconsistent with the MRI findings, enhanced CT showed normal vascular diameter and flow. In addition to these inconsistencies, the “occluded” artery on MRI had shifted and flattened remarkably compared to the axial image of same level on CT (Figure 3). For these reasons, the misleading images were thought to be associated with the MRI protocol.

In our case, a surface coil was placed on the lower abdomen and tightly fixed with a belt as is usual for a pelvic study. The ventral part of surface coil weighs about 2.5 kg. The cause of temporary vascular occlusion was compression of the iliac artery between the enlarged uterus and lumbar vertebrae by tight fixation of the belt and the weight of the coil. If symptoms and image findings do not match, or if there is a large pelvic mass, it may be a flase image, and hence we should confirm with ultrasonography or enhanced CT. It may be better to use the 3D-gradient echo sequence with a Bolus-tracking method (Care bolus, Siemens Healthineers AG, Erlangen, Germany) [6].

## Conclusions

TRC-MRA evaluates hemodynamics precisely based on changes of contrast enhancement of vascular lumens. However, flow alternation can take place depending on patient posture and positioning status, especially in cases with large abdominal or pelvic mass lesions. For patient preparation, coil setting position is important and physicians should be familiar to the potential risk of transient arterial luminal stenosis or even occlusion in patients with a large pelvic mass.
